# Public Health and Residential Care Facilities: Geriatricians' Roles in the COVID‐19 Response and Beyond

**DOI:** 10.1111/jgs.70417

**Published:** 2026-03-31

**Authors:** Meredith Greene, Belinda Tang, Anna H. Chodos, Louise Aronson

**Affiliations:** ^1^ Division of Geriatrics, Department of Medicine University of California San Francisco California USA; ^2^ Department of Medicine, Division of General Internal Medicine & Geriatrics Indiana University School of Medicine Indianapolis Indiana USA; ^3^ Department of Medicine University of California Los Angeles California USA

**Keywords:** assisted living, COVID‐19, geriatric professionals, public health, workforce

## 1 Introduction

The COVID‐19 pandemic highlighted long‐standing emergency preparedness challenges for long‐term care (LTC) settings [1, 2]. Attention to COVID‐19 in LTC focused primarily on skilled nursing facilities (SNFs), however, assisted living and other residential care facilities (RCFs) were also impacted. Despite increasingly serving residents with dementia and other medical vulnerabilities, RCFs pose unique challenges in infection control since they typically do not have medical directors or formal oversight by health departments [3, 4].

In 2020, San Francisco was the first city in the United States to declare a COVID‐19 state of emergency. Soon after, the city's Department of Public Health (SFDPH) established multiple outreach and outbreak teams, including teams focused on RCFs [5]. Three academic geriatricians joined the RCF teams. To help define challenges unique to RCFs and partnerships between geriatrics and public health, we conducted focus groups with key stakeholders from the SFDPH COVID‐19 RCF teams. This manuscript focuses on lessons learned about the geriatricians' roles in the response.

## 2 Methods

### 2.1 San Francisco’s RCF COVID‐19 Response

Two teams comprised the SFDPH COVID‐19 RCF response: (1) the prevention and outreach “SeniorHub” team, and (2) the outbreak management group, “OMG.” These teams interfaced with assisted living facilities (Residential Care for the Elderly or RCFE), RCFs for the Chronically Ill (RCFCI, serving people with HIV/AIDS), and Adult Residential Facilities (ARF, serving adults ≥ 18 years with disabilities). Sites varied significantly by populations served and size, ranging from six‐bed board and care homes to 500+ bed assisted living facilities. Geriatricians' roles included answering clinical questions, conducting site visits, interacting with state oversight agencies, and attending daily team and weekly community partner meetings.

## 3 Participants and Data

We conducted semi‐structured focus groups and interviews via a secure video platform in February 2021 with members of the COVID‐19 RCF teams who managed the first waves of the pandemic in 2020 and early 2021. The interview guide included questions about working with geriatricians, team processes and suggested improvements for future crisis responses (see Supporting Information). Interviews were recorded and transcribed, with transcripts deidentified prior to data analysis.

We first analyzed transcripts inductively through collaborative thematic analysis and then developed and refined a codebook through iterative deductive content analysis. Two coders coded all transcripts. Code discrepancies were reconciled with input from the research team. Procedures were approved by the UCSF Institutional Review Board (IRB#21–35,043).

## 4 Results

Six SeniorHub team members and 12 outbreak management team members participated. Most Senior Hub participants had social work or public health degrees, and most outbreak management participants had nursing degrees. Key themes fit a framework of public health system gaps and geriatricians' roles in helping to address those gaps. Exemplary quotes are provided in Tables 1 and 2.

**TABLE 1:** Exemplary quotes about public health system gaps.

Theme	Subthemes if present	Quote
Lack of aging related clinical knowledge		“And these are the challenges, there's not enough understanding of specific geriatric issues… issues related to impending death and dying and issues related to medical issues later in life, cognitive issues. There's so many things that happen as you get older that people don't understand…”
Lack of long term care sites knowledge		“One of the most difficult things that I found beginning this work is getting even the Department…to say long‐term care is more than skilled nursing facilities. It also includes residential care facilities…But language was ingrained, long‐term care equals skilled nursing facilities…”
		“It's been rewarding but challenging at the same time and I've learned a lot because I came in here not knowing anything about long‐term care facilities but I learned pretty quickly that's a pretty broad term. So that caused me a lot of stress in the beginning of this, my deployment because, you know, I quickly learned that not every assisted living place is the same and has different licensing features to it, I guess. I don't even know if that's the correct terminology. And so, you know, you have your residential care facility for the elderly. You have your adult residential facility.”
	RCF guidance and policies	“I feel like a huge issue has been around policy, too. And, like, guidance documents that are at least I think that I feel, often feel that RCFs and our population are an afterthought, and they aren't included in the initial—I feel like the discussions or first round of policy decisions. And then until something is released and then we go through the guidance documents, or the health order and it doesn't contain any language about our facilities. And then we get questions (From non SNF facilities) and it's—So I think that's often a gap and then we try to then figure out how to address the issues.”
		“For most of the surge and before that, there were very few resources. We were really teaching the regulatory agency about COVID because they are non‐clinical and most of the printed literature and guidelines and such were sort of focused on SNFs. So there was a vacuum and we had to create a lot of stuff, protocols, documents, guidance things, so yes, it's true, the clinical support, the sort of technical—let's call it technical rather than clinical, the technical support for RCFs was much less than SNFs because there wasn't a playbook. They had to adopt the SNF playbook but there's differences, right? Not too many differences. In the end, we think it's very similar, but there were two different regulatory agencies so… In terms of that technical guidance, there was, yes definitely a gap and probably continues to be.”
Limited lifespan approach		“There is a whole section in public health that is standard‐maternal and child health… but there's no sort of adults and older adults…. there's no focus on the rest of the age spectrum… And I think that that can't continue because the population is changing.”
		“I'm not aware if we even at the level public health have basic metrics to follow the health and well‐being, medically, socially, for old people—I don't even know if we have those but we are obviously failing on all those.”
Siloes between public health and aging services		“…there needs to be an opportunity to avoid issues like the siloing, that everybody who has a stake in San Francisco's residential care facilities, are at the table… what can we do to improve the facilities and doing that in tandem with the Health Department and Department of Social Services.”
		“So it was the first time really the [San Francisco] Department of Public Health has worked with the California Department of Social Services and their Regional Office around these residential care facilities. I mean, the Department of Public Health in my knowledge has—and I work in disaster preparedness, we didn't really have a good way of reaching out to these facilities and didn't quite know how many there were in the past.”

Abbreviation: RCF, residential care facilities.

**TABLE 2:** Additional quotes on how geriatricians contributed to public health response.

Theme	Subtheme	Quote
Aging expertise	Clinical	They provide clinical expertise and served as subject matter experts within our small team and for the larger public health department. They bring a wealth of knowledge and experience with residential care facilities, older adults and adults with chronic illness.
		They've all worked with the clients that we have had…they work with, these residents, who live in these places and so they're all very familiar with, you know, the many issues that accompany these residents.”
		“the geriatricians brought awareness to the symptoms/presentations of COVID‐19 in older adults that I don't see listed anywhere…For example, I often advise facility administrators to watch out for potential COVID 19 symptoms such as a > 2 degree temperature change even if under 100 degrees F, loss of appetite, sudden falls or unusual behavior from their baseline. People were always shocked to hear that because they haven't heard it from anywhere else.”
	Clinical‐disability care	“…they are geriatricians but a lot of the clients that we're working with in these places are not necessarily seniors. You know, they're, you know, physically challenged or, you know, have mental issues and so they're not all over 60. And so they were really helpful with all of them, not just the seniors”
	Clinical‐dementia care	“It's been super helpful, you know, learning about older illness and all of that stuff. You know, they also have a lot of expertise in dementia care which we certainly don't have, so that's been really nice also. Yeah, the dementia piece. “
	Holistic approach	“They have a different perspective because we were really focused on infection control and then we had these geriatricians come in and they are like what about their emotional state, what about these other things that may be inflicting them, dementia, or all these other things that were not on our radar because we were just thinking infection control. It really helped to broaden our view on how to respond.”
		“And I think geriatricians by nature of their work are very holistic. Even though they are holistic in‐terms of a single patient, it's so closely connected to public health in that they think about systems of care. They have to think about systems of care. So there is almost a very natural connection between public health and geriatrics and imagine if we had metrics… even just being aware of the fact that so many elders are living alone or there's ten elders to one homecare worker in a facility like this. Just that awareness, it's not what's sexy and exciting for popular culture but—though, I feel like that it would contribute to a more compassionate society, I hope.”
Knowledge residential care sites		“But like, I think like, there's a clear, like understanding of like, from geriatricians specifically, like what implementation challenges would look like in terms of a public health response to like an outbreak. So that's really important. So there's a lot of like, on the ground experience.”
		“I would've had much less sense of the overall landscape of eldercare if not for<Anonymized> and her feeding me a few resources along the way and her impressions just in general and probably I don't even realize how much I learned about that stuff through her presence on the larger team, but she was key in having a geriatrician who is interested in the landscape and the big picture of geriatrics was very helpful to understand more about like how many RCFEs are in the city, how do they get funded, what are their margins like, things like that.”
Advocacy		“Their expertise in geriatrics, COVID‐19 research, and policy implications on older adults has been invaluable… For example, they have identified communications and policies that have ageist implications and have escalated these issues within the public health department.”
		“But I guess what I mean—I don't mean medically—I mean more socially. The way that they advocated for them as people was different from the way that like I was probably approaching it just in terms of advocating for them simply as sort of like frail little bags of cells and chemicals. They were thinking of them like—they were advocating for them as functioning humans, like members of society in a more holistic way and also calling out the ageism that was very present in materials and in the way we talked in policies and the way we have—the way that safety gets prioritized over autonomy in a really consistent way in all these discussion and the ageism inherent in that and the way that tradeoff is often unquestioned, and it's like who is making that choice on whose behalf and who gets to participate in it. I think they were raising those issues, which I was—I think of myself as a person who is already trying to do those kinds of things and yet I needed a lot of reminders and kind of shown how to do it along the way and they were there to do it and I really appreciated that.
		Highlight the population that we will all be part of (hopefully), seniors are the future “we” so we must keep senior populations in mind and at the table when planning, developing, implementing public health programs and efforts. Geriatricians can also highlight becoming older as a normal stage in life that must garner the same attention as infant, youth and adult public health, healthcare access, policy, and design considerations.
		And then also I think that they also were really good in addition to their professions just at communicating and establishing relationships with people in a way where they were able to kind of massage policies through. I think that kind of work is really, really hard.
		They were, I felt like liaisons because they were pushing for better resources for that community and they were able to relay information that the community was requesting to the public health department.
Interprofessional approach		“… coordinating the resources that are available, you know, they have an understanding of like, who the key players are. So again, that's like CDSS [California Department of Social Services], and like, who those people are, and how to, maneuver, you know, getting resources and things like that, how to advocate for that, and how to do that effectively.”
		“There was absolutely no sense of hierarchy or entitlement or, you know, feeling above. It was just so collaborative. They're such collaborative clinicians and docs. And that was really very, very—And maybe that's specific to geriatricians, I don't know.”
		“And then also I think that they also were really good in addition to their professions just at communicating and establishing relationships with people in a way where they were able to kind of massage policies through. I think that kind of work is really, really hard.”

### 4.1 Public Health System Gaps

#### 4.1.1 Theme 1: Lack of Aging Knowledge

Team members were unfamiliar with atypical presentations of infections, complexity management, and care options for older adults whose health warranted care in RCFs.

#### 4.1.2 Theme 2: Lack of Familiarity With RCFs

Many COVID‐19 policies assumed all LTC facilities had similar resources as SNFs—for example, that RCFs could routinely monitor vital signs.

#### 4.1.3 Theme 3: Partial Lifespan Approach

Team members voiced disappointment over the limited public health services for older adults compared to children and adults.

#### 4.1.4 Theme 4: Limited Collaboration Between Public Health and Aging Services

Networks between SFDPH and RCFs were not well established. The teams assembled de novo facility and contact lists, including key players in the RCF regulatory space.

### 4.2 Geriatricians Helped Address the Gaps

#### 4.2.1 Theme 1: Expertise in Aging and Disability

Expertise in the unique symptom presentations of older adults helped refine the response. Geriatricians' holistic view of physical, mental, and social health expanded public health workers' perspectives. For example, while strict lockdown policies restricted COVID‐19 spread, they also led to declines in mental health and physical function.

#### 4.2.2 Theme 2: Knowledge of RCFs

The geriatricians educated staff about the range of LTC facilities, regulations, and capacities.

#### 4.2.3 Theme 3: Aging Advocacy

The geriatricians advocated for older adults with SFDPH leadership and helped public health professionals broaden their approach to encompass all stages of life.

#### 4.2.4 Theme 4: Interprofessional Collaboration

Interviewees praised the geriatricians for their collaborative nature and aid in breaking down siloes between aging and public health services.

## 5 Discussion

Our study offers ideas for future public health‐geriatrics partnerships. SFDPH's creation of interdisciplinary teams and recruitment of geriatricians for those teams strengthened the city's COVID‐19 response in RCFs. Three academic geriatricians working within SFDPH helped address RCF knowledge gaps, bridged siloes between public health and aging services, assisted with public communication, and advocated for older adults and people with disabilities.

Other articles highlighting RCF administrators' perspectives of the COVID‐19 response echoed our findings of care silos and unfamiliarity with RCFs [6, 7]. Public Health staff in Westchester NY and Houston similarly reported the inadequacy of traditional symptom screenings, especially in memory care [8], and considerable variation in preparedness and infection control interventions among facilities [9]. With a rapidly expanding RCF market, high turnover of facility staff, and shrinking public health budgets, these concerns remain relevant. At this historical moment, geriatricians may be well positioned to provide guidance and expertise to RCFs while also taking advantage of novel opportunities for professional growth and impact.

Our study had limitations. We present the experiences of one health department in an urban area with existing public health, academic, and health care delivery system partnerships. The geriatricians involved in this study initially volunteered their time (later, they became paid consultants), and their impact may reflect personal motivations and interests. Given the unprecedented crisis, public health staff and other city employees were pulled into different departments, and knowledge may not reflect usual practices.

Applying lessons learned from COVID‐19 will be critical in improving emergency preparedness for RCFs going forward. Our findings support the need for “age friendly public health systems” [10] but also suggest ways for geriatric health professionals to support public health. With increasing public mistrust, it is especially important that geriatric health professionals consider investigating and supporting the expansion of geriatric expertise into RCFs and public health.

## Author Contributions

Meredith Greene, Louise Aronson, and Anna H. Chodos designed the study. Meredith Greene, Belinda Tang, and Anna H. Chodos conducted the implementation of the study. Meredith Greene and Louise Aronson analyzed the data. Meredith Greene, Belinda Tang, and Louise Aronson wrote the manuscript with consultation from Anna H. Chodos. All authors provided critical feedback and helped shape the research, analysis, and manuscript.

## Funding

This work was supported by the National Institute on Aging (K76AG064545).

## Disclosure

Dr. Greene receives funding from the NIA (K76AG064545). Portions of this paper were presented as a poster presentation at the American Geriatrics Society Annual Conference in 2021.

## Conflicts of Interest

The authors declare no conflicts of interest.

## Supporting information


**Data S1:** Supporting Information.
