# Supplementary material for: Public Health and Residential Care Facilities: Geriatricians' Roles in the COVID‐19 Response and Beyond
Source: J Am Geriatr Soc. 2026 Mar 31;74(7):2124–9. doi: 10.1111/jgs.70417 (PMC13418571; doi:10.1111/jgs.70417)
Supplement: Supplementary file 1 — Data S1: Supporting Information. [file JGS-74-2124-s001.pdf]

Supplementary Materials
S1. Interview Guide

Prevention/Outreach:
We are going to talk about the prevention efforts and outbreak response in SF Residential Care Facilities/Assisted Living Facilities. We want to hear your thoughts about what worked well and what didn't.

What was the initial process for outreach and prevention?
When did that change and why?

When you changed course, why? What didn't work? What challenges was the new system meant to address?
Prompts: barriers/facilitators to different topics (from AGS policy)
e.g. What barriers to infection control were identified on site visits?
-workforce
-testing and contact tracing
-isolation and quarantine, transitions (ER/hospital/facility/ ADL needs)
-supply chain
-medical/non-medical model/compared to SNF

What worked well in ALFs?

What would you do differently in assisted living pandemic response if you had to do it over again? What were some of the important lessons learned from your involvement in response.

What would you advise/what suggestions do you have for others who are doing similar work or thinking about doing similar work? For future pandemics?

What would be the ideal system/response? What are some of the challenges or barriers you currently see to having this ideal system? What are some of the systems or structures currently in place that might make this system possible?

Outbreak:
What were you most surprised by with the effort to respond to outbreaks in RCFEs?
What were common challenges seen on site visits?

- We realize that staffing/ team organization were major challenges, but we would like to focus on the facilities themselves.

Did you notice any differences or unique challenges in your team's response to these sites versus the other OMG sites/types of congregate settings?

Were there things that went really well that other people should try to replicate or learn from?

If you had to start over, with what you have learned, what advice would you give to someone or what would you do differently this time around?

The American Geriatrics Society identified some **key areas** for cities/counties to focus on in support these sites through COVID. Do these look right to you? Are there key areas you would add?

- Supply chain: e.g. improve this through the Defense Production Act
- COVID-19 Testing and Contact Tracing
- Safe transitions (to emergency room, etc.)
- Infection control
- Workforce (paid leave, supporting the workforce)

What do you think about these now that you have responded to many outbreaks?

Roles and value added by geriatricians

What was your experience having a geriatrician on the team? (seniorhub team)

What ways did the geriatrician(s) contribute to the team? To the overall response?
--what knowledge, experience did they bring? (ask specifically about knowledge sites if not brought up)
What can geriatricians contribute to public health
